# Environmental Stress Affects DNA Methylation of a CpG Rich Promoter Region of Serotonin Transporter Gene in a Nurse Cohort

**DOI:** 10.1371/journal.pone.0045813

**Published:** 2012-09-28

**Authors:** Jukka S. Alasaari, Markus Lagus, Hanna M. Ollila, Auli Toivola, Mika Kivimäki, Jussi Vahtera, Erkki Kronholm, Mikko Härmä, Sampsa Puttonen, Tiina Paunio

**Affiliations:** 1 Public Health Genomics Unit, National Institute for Health and Welfare, Helsinki, Finland; 2 Finnish Institute of Occupational Health, Helsinki, Finland; 3 Department of Epidemiology and Public Health, University College London, London, United Kingdom; 4 Department of Chronic Disease Prevention, National Institute for Health and Welfare, Turku, Finland; 5 Department of Psychiatry, Helsinki University Central Hospital, Helsinki, Finland; Università di Napoli Federico II, Italy

## Abstract

**Background:**

Shift-working nurses are exposed to a stressful work environment, which puts them at an increased risk for burnout and depression. We explored the effect of environmental stress on serotonin transporter gene (*SLC6A4)* promoter methylation among nurses from high and low work stress environments.

**Methodology:**

Using bisulfite sequencing, we investigated the methylation status of five CpG residues of a CpG-rich region in the promoter of *SLC6A4* by comparing female shift working nurses from a high work stress environment (n = 24) to low work stress environment (n = 25). We also analyzed the association of *5-HTTLPR* polymorphism at 5′ end of *SLC6A4*. Work stress was assessed by the Karasek’s Model and possible signs of burnout or depression were measured by the Maslach Burnout Index General Survey and Beck Depression Index. Methylation levels were assessed by bisulfite sequencing of DNA extracted from peripheral blood leucocytes. Restriction enzyme treatment followed by standard PCR was used to identify *5-HTTLPR* genotypes.

**Principal Findings:**

We found that nurses in the high stress environment had significantly lower promoter methylation levels at all five CpG residues compared to nurses in the low stress environment (p<0.01). There was no significant interaction of 5-HTTLPR genotype and work stress with methylation (p = 0.58). In unadjusted (bivariate) analysis, burnout was not significantly associated to methylation levels. However, when mutually adjusted for both, burnout and work stress were significant contributors (p = 0.038 and p<0.0001 respectively) to methylation levels.

**Conclusions:**

Our findings show that environmental stress is concurrent with decreased methylation of the *SLC6A4* promoter. This may lead to increased transcriptional activity of the gene, increased reuptake of serotonin from synaptic clefts, and termination of the activity of serotonin. This could present a possible coping mechanism for environmental stress in humans that could eventually increase risk for disturbed functional capability and experience of depressed mood in long-term stress.

## Introduction

Chronic work stress induces adverse emotional and physical responses, which are triggered by perception of work demands that exceed the person’s capacity and ability to cope [1]. Such stress has a negative impact on job performance and is now becoming a leading cause of work absence in western society, increasing economic pressure particularly in the public sector. Our biological system strives to maintain a state of homeostatic equilibrium to avoid prolonged, chronic stress that can be harmful to our body [2]. Chronically persisting and uncontrollable environmental stress can potentially lead to more severe psychosocial syndromes such as burnout and depression [3].

Research on mechanisms underlying stress adaptation and stress susceptibility have received greater attention in recent years as we are beginning to understand that environmental factors and genetic variation are not the sole contributors to behavioral and emotional illnesses. Some individuals seem to be able to cope with stress better than others and it is assumed that this is partly influenced by epigenetic mechanisms [4]. DNA methylation has been suggested to be one of the possible mechanisms to mediate the response of individuals to stress [5].

In humans, DNA methylation occurs, almost exclusively, through covalent modification of DNA where methyl groups are coupled to cytosine residues of CpG dinucleotides. DNA methylation has been shown to associate with variation in gene expression [6], whereby serving as a possible mechanism for response to extracellular events.

Several published studies on stress-related outcomes have proposed a relationship between environmental stress and epigenetic changes. DNA methylation variation has been linked to early life stress in a rodent model [7], [8] and later to the serotonin transporter gene (*SLC6A4*) in humans [9]. It has also been reported to be affected by child abuse [10] and is believed to be a mechanism linking childhood sex abuse to increased risk for antisocial personality disorder [11]. Risk for posttraumatic stress disorder has been shown to be modified by methylation levels [12]. Individuals with a lifetime history of major depression were found to have increased overall methylation levels [13]. Furthermore, there is evidence that pharmacological interventions have the potential to reverse environment-induced modification of epigenetic states [8], [14], [15].

*SLC6A4* is of particular interest in the context of stress-related outcomes and epigenetic changes [9]–[12]. Methylation at this locus is believed to be an important contributor to vulnerability to neuropsychiatric illnesses. *SLC6A4* is an integral membrane protein mainly in the central and peripheral nervous systems that transports serotonin (5-HT) from synaptic spaces into pre-synaptic neurons and serves to regulate emotional aspects of behavior [16]. This transport process by *SLC6A4* terminates the action of serotonin. Reduced 5-HT levels can possibly increase susceptibility for a life time risk for depression [17].

A functional polymorphism in *SLC6A4* gene-linked polymorphic region, termed *5-HTTLPR*, has been reported to affect mood and behavior in humans [18]–[20] in particular when combined with recent or early stressful life events [21], [22]. The short (S) allelic variant of *SLC6A4*, as opposed to the long (L) variant with a 44-bp insertion [23] has been associated with decreased mRNA transcription [13], [24]. A single nucleotide polymorphism (rs25531) inside the long allele (Lg or La) further modifies expression of the gene so that the Lg allele is functionally similar to the S allele [25].

In this study, we sought to assess the association of environmental stress, measured as work stress, with DNA methylation at five CpG residues in the *SLC6A4* promoter in nurses from high and low work stress environments. Furthermore, we analyzed the *5-HTTLPR* length polymorphism and its possible association to CpG methylation in the promoter region.

## Results

### Burnout and Depression Scores in High and Low Work Stress Groups

Our study sample comprised of female nurses from contrasting work environments (high and low work stress, n = 24 and 25 respectively) initially derived from a large cohort of 5615 female health care professionals [26]. Works stress was defined according to Karasek’s Model [27] so that high work stress implicated high demands and low control, while low stress was defined by a combination of low demands and high control. Division into high and low work stress environments was based on grouping the wards based on mean scores of all responses, using median split to identify high and low stress extremes in stress distribution. To increase contrast between the comparison groups, the nurses who belonged to the quartile with least work stress in the high stress group and most stress in the low stress group were excluded.

To assess individual burnout, each subject answered the Maslach Burnout Index General Survey (MBI-GS). The mean MBI-GS scores were 1.3(±0.76) and 0.72(±0.49) in the high and low stress groups respectively (Table 1). Half (n = 12) of the 24 subjects in the high work stress group reported at least moderate burnout symptoms (MBI-GS scores >1.5). There was no reported burnout in the low work stress group of 25 nurses (MBI-GS scores <1.5). The difference in mean MBI-GS scores between the high and low stress groups was statistically significant (p = 2.3×10^−5^) as per t-test, which indicates higher individually experienced burnout in the high work stress environment. 12 subjects in the high work stress group reported mild depression according to the Beck Depression Index (BDI scores 10–18) while two subjects scored moderate to severe depression (BDI scores >18). Only three subjects reported mild depression in the low work stress group and none scored moderate to severe depression. The mean BDI scores were 8.36(±6.31) and 4.37(±3.36) in the high and low work stress groups respectively (Table 1). Also, as expected, depression was significantly associated with work stress (p = 2.0×10^−3^) as per t-test.

**Table 1:** Demographic data of the nurse groups by work stress.

Work Stress Environment	N	Age	MBI-GS	BDI
High work stress	24	49 (±6.02)	1.33 (±0.760)	8.36 (±6.31)
Low work stress	25	48 (±6.78)	0.723 (±0.487)	4.37 (±3.36)

### Methylation Levels

**Figure 1:**
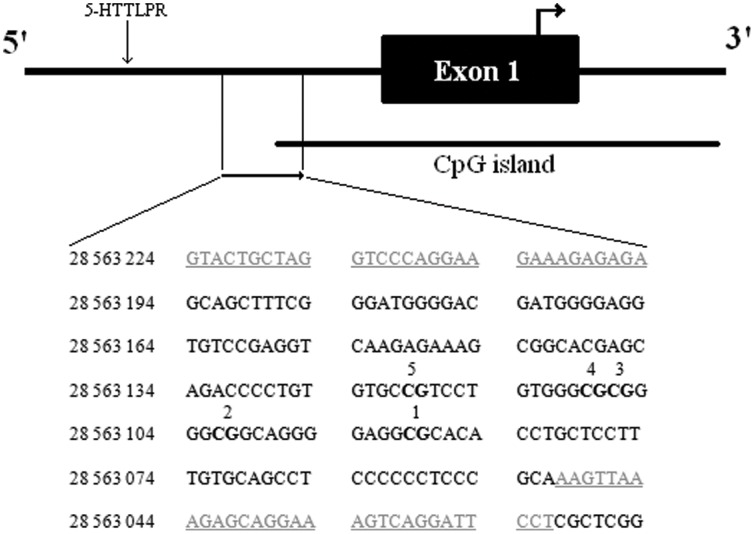
Representation of the *SLC6A4* gene promoter region for bisulfite sequencing analysis. Analyzed CpG sites are bolded and numbered from 1 through 5. Coordinates are based on the GRCh37 build (NCBI Reference Sequence: NC_000017.10).

**Figure 2:**
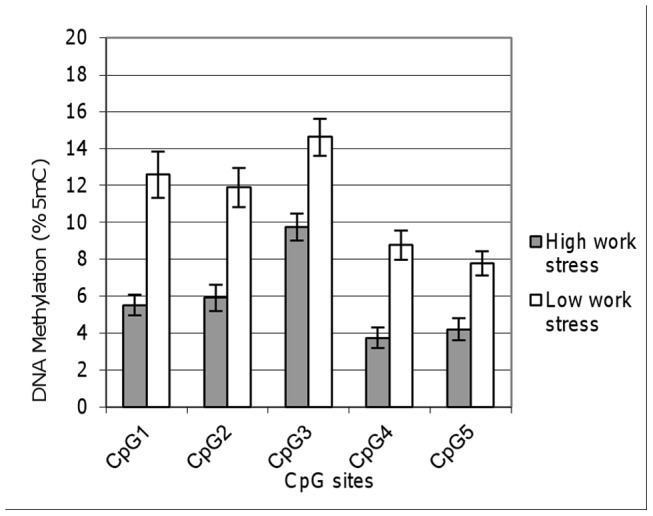
Methylation in the promoter of *SLC6A4* at five CpG sites in high (grey) and low (white) work stress environments. Coordinates for each residue are 28 563 120 (CpG5), 28 563 109 (CpG4), 28 563 107 (CpG3), 28 563 102 (CpG2), and 28 563 090 (CpG1) as per GRCh37 build (NCBI Reference Sequence: NC_000017.10). Differences between work stress environments were significant as per t-test (p = 7.10E–06, p = 2.50E–05, p = 0.000292, p = 4.37E–06, and p = 0.000289 respectively). Standard errors are indicated.

We performed bisulfite sequencing of part of the promoter region of *SLC6A4* (Figure 1), including 5 CpG residues, among nurses from high work stress (n = 24) and low work stress (n = 25) environments. Coordinates for each CpG residue were 28 563 120 (CpG5), 28 563 109 (CpG4), 28 563 107 (CpG3), 28 563 102 (CpG2), and 28 563 090 (CpG1) as per GRCh37 build (NCBI Reference Sequence). The initial focus of this study was to compare methylation levels of five CpG residues in the promoter of *SLC6A4* in relation to work stress using bisulfite sequencing. Using t-test, we saw significantly lower methylation levels in the high work stress group across all five CpG –residues (CpG1: 5.5±2.7, CpG2:5.9±3.5, CpG3: 9.8±3.7, CpG4: 3.7±2.8, CpG5: 4.2±3.0) in comparison to low work stress (CpG1: 13±6.7, CpG2: 12±5.7, CpG3: 15±5.4, CpG4: 8.8±4.3, CpG5: 7.8±3.5) (p<0.01 for all residues, Figure 2). Methylation results were then validated in 9 pairs of nurses matched by age from high- and low work stress environments using the Human Methylation 450k BeadChip (Illumina Inc.). While variation in methylation was smaller as compared to the bisulfite sequencing in all three sites (CpG1, CpG4 and CpG5) included in the BeadChip, differences in methylation levels remained in the same direction in most cases of the matched pairs (7 out of 9 for CpG1 and CpG4 and 9 out of 9 for CpG5), evidencing for a good coherence between these two methods (p = 0.998 from goodness of fit using chi-squared test).

We then tested correlations between individual methylation levels in all five CpG residues. All methylation levels were highly correlated (β>0.5, p<0.01 for all residues). Using a structural equation measurement model, we tested whether these correlations could be explained by a single latent factor. The single factor model was statistically accepted (df = 5; χ2 = 4.1; p = 0.537). This was considered as a justification for constructing a single weighted sum score for methylation levels. A principal component analysis (PCA) revealed 5 components of which the first one explained 79% of the total variance of all CpG residues. A sum score for methylation was then calculated by multiplying the values for each individual CpG residue by its factorial loading obtained in the first component of PCA. These weighted values were then summed up to create a weighted sum score, denoted as METsum throughout this study.

The mean values for METsum in the high and low work stress groups were 25.7±10.6 (95% confidence interval 21.2–30.1) and 50.7±21 (95% confidence interval 42.0–59.4) respectively (see Table S1). Based on confidence limits, the differences between the two work stress groups were evident. The mean METsum in the total sample was 38.4±20.8. In order to determine the effect size between work stress groups, we calculated the Cohen’s d for the difference between means [(50.7–25.7)/20.8 = 1.2]. The effect size of 1.2 indicates that the difference is notable.

**Figure 3:**
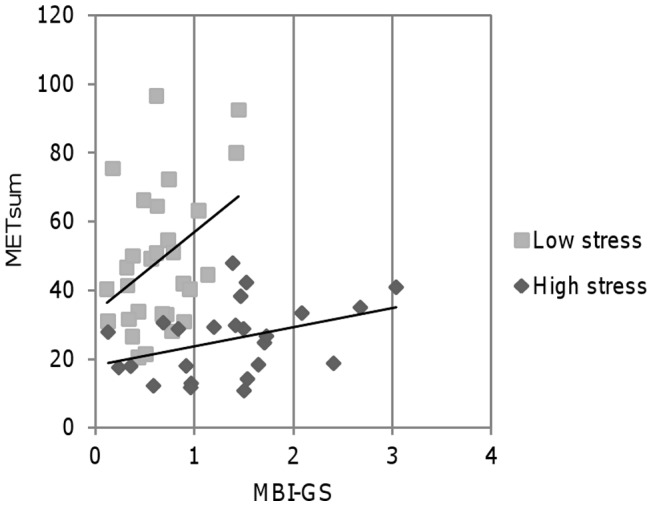
Methylation levels based on burnout scores in high and low work stress environments. 12 nurses in the high stress environment exhibit moderate burnout (MBI-GS>1.5). Nurses in the low stress environment report no signs of burnout (MBI-GS<1.5).

We then performed a multifactorial analysis of covariance to assess the association of METsum (as the dependent variable) with work stress environment, burnout, work control, work demand, age, and *5-HTTLPR* (as explanatory variables). When the main effects of all explanatory variables were included into the model, only work stress was statistically significant and independently (F = 5.7; p = 0.022) associated with METsum (see Table S1). However, when non-significant effects were eliminated one by one from the model, it was found that in the final model both work stress (p<0.0001) and MBI-GS (p = 0.038) were independently associated with methylation levels. Together they explained 43% of the total variance of METsum (R^2^ = 0.43, p<0.0001). When the effect of work stress environment was adjusted, burnout was positively associated with methylation levels. In other words, in stratified analyses separately in high and low work stress environments, higher burnout scores were associated with higher methylation levels (Figure 3). Because of a small number of observations, these stratified analyses did not reach statistical significance (p = 0.062 in high work stress environment and p = 0.065 in low work stress environment).

### *5-HTTLPR* Genotype

Next we examined the distribution of *5-HTTLPR* polymorphism and its association with methylation and MBI scores. Mean METsum levels for the La/La, La/S and S/S genotypes were 35.3, 36.5 and 49.9, respectively (Table 2). There was no significant association between *5-HTTLPR* and METsum (p = 0.25), *5-HTTLPR* and work stress (p = 0.067) or *5-HTTLPR* and burnout (p = 0.50). We then tested interactions for the genotype and work stress or burnout in a multivariable model but found no statistically significant evidence for these interactions (p = 0.58 and p = 0.082, respectively). Effect sizes between La/La and S/S, La/S and S/S, and La/La and La/S were 0.73, 0.57, and 0.06 and their minimal detectable effect sizes were 1.03, 10.09, and 1.02, respectively.

**Table 2:** Distribution of the 5-HTTLPR genotype.

Genotype	La/La	La/S	S/S
N (%)	21 (39.6%)	19 (35.8%)	9 (17.0%)
Mean burnout scores (SD)	0.889 (±0.764)	1.02 (±0.604)	0.951 (±0.499)
Mean depression scores (SD)	6.48 (±6.32)	6.05 (±4.94)	6.89 (±4.34)
METsum (SD)	35.3 (±16.2)	36.5 (±22.3)	49.9 (±25.3)

## Discussion

Our initial results showed that DNA methylation levels in the promoter region of *SLC6A4* varied between high and low work stress environments among female nurses. Individuals working in a high work stress environment have 21–65% lower levels of methylation compared to individuals in a low work stress environment. Methylation was not associated with *5-HTTLPR* genotype. However, we observed 1.4-fold higher methylation levels among individuals carrying the S allele on both of their chromosomes than among L/L individuals, which is concurrent with previous studies showing higher *SLC6A4* methylation in blood cells of S-allele carriers [28], [29]. We found no evidence in our data that the *5-HTTLPR* polymorphism would contribute to methylation, burnout or environmental stress, which contradicts with earlier interaction studies [21], [28], but our study was underpowered to detect such interaction as suggested by the calculated effect sizes and minimal detectable effect sizes.

In a recent study, hundreds of methylation linkage disequilibrium (LD) blocks were identified in over 2000 CpG islands [30]. These methylation LD blocks not only cover imprinted genes and X-chromosome regions in females due to X-chromosome inactivation, but they are also found among other genes. Methylation levels in all five CpG residues of the present study, located at close vicinity to each other in a stretch of 30 bp, were highly correlated and shared a single latent factor according to structural equation model. Consequently, a summed methylation score (METsum) from the principal component analysis was used in subsequent analyses in order to represent general methylation level at the region of interest.

In most cases methylation at gene promoters leads to silencing of the gene itself. An *in vitro* study shows that *SLC6A4* promoter methylation results in decreased levels of mRNA, although this effect appears stronger when *5-HTTLPR* polymorphism is taken into account [13]. The protein encoded by *SLC6A4* is responsible for the reuptake of 5-HT from the synaptic clefts and higher levels of *SLC6A4* expression will invariably lead to increased reuptake of 5-HT, which decreases the efficiency, magnitude and duration of 5-HT. In the context of this study, we hypothesize that hypomethylation of *SLC6A4* presents an adaptational mechanism for stress. While this adaptation is physiological and initially serves to maintain the individual’s best possible functional capacity during stress, it might, eventually, increase risk for functional disturbances, such as decreased cognitive ability and depressed mood, simultaneously with failure of other coping mechanisms. Interestingly, relative lack of serotonin in brain is one of the major hypotheses of depressive disorder, and serotonin transporter is known to be one of the major targets of many antidepressants, including the selective serotonin reuptake inhibitors [31].

All nurses in the high stress environment had been working in the same ward for at least 3 consecutive years with relatively low absenteeism and they showed no signs of clinical depression, despite being exposed to a chronic stressful environment. Environmental stress resulted in decreased methylation of the *SLC6A4* promoter in blood leucocytes. In theory, if this occurred also in neurons, it would lead to decreased amounts of extracellular 5-HT in the synaptic clefts, presenting the body’s message to ‘slow down’.

In terms of serotonin biology outside the central nervous system, there are a myriad of other effects of serotonin indicating that it is more than a neurotransmitter for the modulation of mood. Serotonin regulates a wide range of physiological processes in most human organs such as cardiovascular function, bowel motility, intestinal peristalsis and secretion, platelet aggregation and bladder control [32], which in turn explains why serotonergic drugs affect several physiological processes at multiple levels and different mechanisms in addition to effects on mood and cognition. Indeed, a recent study shows that acute serotonin depletion has little effect on mood in normal healthy individuals [33].

Some limitations in our study should be noted. First, measurement of methylation levels is semi-quantitative by its nature, prone to artifacts caused by the amplification process, and thus requires validation by other, non-PCR based, methods. We validated our findings using the Human Methylation 450 k BeadChip (Illumina Inc.) and found smaller variation in general methylation. However, differences in the matched pairs of nurses remained significantly similar when the two methods were compared. Furthermore, we observed some cytosine (C) background noise for forward sequencing for some samples in our initial tests. By incorporating 20 bp overhangs, that contained C and G nucleotides, at 5′ ends of primers, we were able to improve sequence quality and reduce cytosine background significantly [34]. Accurate primer optimization has also been reported to overcome bias in bisulfite methylation analysis [35].

Second, results from peripheral blood leucocytes may not directly be extrapolated to the human brain. However, stress arguably affects the entire body at many levels and, as previously mentioned, serotonin affects a vast range of other functions in that system. The most commonly used source of DNA in *SLC6A4* methylation studies is blood tissue. In these studies, DNA is either extracted from peripheral blood leucocytes or from lymphoblast cell lines. Finally, we also acknowledge the heterogeneity of our sample as whole blood samples contain a mixture of various cells that exist in the blood circulation.

Third, our small sample size (n = 49) is limiting in terms of statistical power. The original sample size (n = 95) was significantly higher, but it was important to keep a strict selection criteria to rule out the possible effects of smoking, alcohol consumption and medication. This can be viewed as a strength in this study, compared to many others, as smoking [36] and alcohol consumption [37], [38] have been shown to affect DNA methylation. Additionally, our sample was drawn from a large starting cohort (n = 5615) enabling us to design a clear contrast in terms of environmental stress based on the well-known Karasek Model.

In conclusion, we found that DNA methylation levels at the promoter CpG upstream of *SLC6A4* are significantly lower among female nurses working in a high stress environment compared to female nurses working in a low stress environment. In addition, subjective symptoms of burnout were associated with higher methylation levels when the effect of work stress environment was taken into account. *5-HTTLPR* does not interact with work stress and methylation, which emphasizes the notable relationship between methylation and environment in the formation of the individual’s phenotype.

## Materials and Methods

### Ethics Statement

This study was conducted according to the guidelines of the Helsinki declaration, and the study protocol was approved by the Ethics Committee of the Finnish Institute of Occupational Health.

### Subjects

Clinical data was obtained from a Finnish cohort comprising of female health care professionals, mainly nurses, midwives and nursing assistants. 422 individuals were recruited from a total of 5615 health care professionals that were part of a Finnish Public Sector Study [26]. This group represents the entire personnel of 21 Finnish public hospitals. These subjects were classified into lowest and highest quartile on work stress as per Karasek’s model [27]. Inclusion criteria was age between 30–60, mother tongue Finnish, BMI under 35, at least 3 years of work experience in the same ward and no greater than 6 months of absenteeism from work during the past 3 years. 99 participants took part in laboratory measurements. There were a total of 95 successful laboratory measurements for further analysis. All laboratory assessments were performed blind to ward work stress status.

Exclusion criteria for the current study were use of medication affecting cognitive functions (Sepram), use of hormonal medication (dostinex, estrogen), and other significant medication (Tamofen for cancer medication), heavy smoking (8 individuals with reported daily smoking for at least 10 consecutive years) and high alcohol intake (1 individual with an intake of 4 or more doses of alcohol over 4 times a week). Individuals, who had missing data or who did not respond to questions referring to any of the above criteria were also excluded (19 individuals). After exclusion criteria, a total of 67 nurses were selected for analysis. Peripheral blood samples were obtained from well-rested individuals. Written consent was obtained from all participants. A total of 49 subjects (73%) were bisulfite sequenced and included in the final analysis for this study. A detailed flow chart for sample selection is provided (Figure S1).

### Work Stress, Burnout and Depression Assessment

From the baseline 5615 female health care professionals, potential subjects (n = 422) were chosen to respond to the Karasek’s Job Content Questionnaire (JCQ). This was based on two consecutive questionnaires of work stress in 2004 and 2008. Three questions assessed job demand and 9 questions assessed job control. Responses to all questions were given on a 5-point scale (completely agree, somewhat agree, not agree/neither disagree, somewhat disagree, completely disagree). The division into high and low work stress groups was based first on grouping the wards with at least 5 respondents according to the mean score of survey responses to the job demand and job control scales at ward level, using median split to identify high stress (high demands and low control) and low stress (low demands and high control) wards (Figure S2). Using these cut-off points, the nurses from the wards were identified, who belonged to the same high and low stress groups also based on their individual mean score values of job demands and job control. Finally, to increase contrast between the comparison groups, nurses who belonged to the quartile with least stress in the high stress group and most stress in the low stress group were excluded.

To assess burnout in our study sample, each subject took the Maslach Burnout Inventory General Survey (MBI-GS). MBI-GS is a modified version of the original MBI to measure levels of burnout in occupations that involve working closely with people [38]. The survey covers all the three components of burnout: exhaustion (EX), cynicism (CY), and professional efficacy (PE). Subjects scoring higher than 1.5 in the MBI-GS were considered to have at least moderate burnout.

Depression was measured using the Beck Depression Inventory (BDI) [39]. The questionnaire is widely used to screen depression in clinical practice and in community samples [40]. Subjects scoring between 10 and 18 were considered to have mild depression while scores of 19 to 29 represent moderate to severe depression.

### Site-specific DNA Methylation

We isolated DNA from peripheral blood leucocytes using QIAGEN Autopure (Qiagen). CpG methylation status of the CpG-rich region in the *SLC6A4* gene promoter was investigated by bisulfite sequencing [41]. The region of *SLC6A4* that was included in the analysis (Figure 1) has been previously shown to be differentially methylated in infants exposed to prenatal maternal depressed mood [9].

500 ng of genomic DNA from leucocytes was treated with sodium bisulfite using the EZ DNA Methylation-Gold Kit (Zymoresearch) following the manufacturer’s protocol. The region of interest was amplified by standard PCR from bisulfite-treated DNA using AmpliTaq Gold Hot Start Polymerase (Applied Biosystems) based on previously published primers [9] with slight modifications in region coverage as a necessity to produce good quality sequence with direct sequencing. SLC6A4F: gtattgttaggttttaggaagaaagagaga and SLC6A4R: aaaaatcctaactttcctrctctttaactt (Figure 1).

Overhanging tails in the 5′ end were designed for both forward and reverse primers that match with common T7 (taatacgactcactataggg) and T3 (attaaccctcactaaaggga) sequencing primers respectively. Cycling conditions were 95°C for 11 minutes followed by 40 cycles of 95°C for 30 seconds, 60°C for 30 seconds, and 72°C for 40 seconds with a final extension of 10 minutes at 72°C. PCR products were verified randomly on a 2% agarose gel (GellyPhor, Euroclone). The remaining PCR product was purified using the Quickstep 2 PCR Purification Kit (Edge Bio).

Cycle sequencing was performed with the BigDye Terminator version 3.1 Cycle Sequencing Kit (Applied Biosystems) using T7 and T3 sequencing primers. Sequencing was performed by capillary sequencing (Finnish Institute for Molecular Medicine). A similar method has been previously described [42].

Methylation percentage at each CpG site was quantified manually using the Sequence Scanner version 1.0 (Applied Biosystems) by two independent examiners (JA and AT) blind to the sample identification codes. The peak heights of C versus the combined heights of C + T peaks (C/C+T) at each CpG locus were calculated as a percentage [Lewin et al. 2004]. The five CpG locations were 28 563 120 (CpG5), 28 563 109 (CpG4), 28 563 107 (CpG3), 28 563 102 (CpG2), and 28 563 090 (CpG1) as per GRCh37 build (NCBI Reference Sequence: NC_000017.10). The success rate for DNA amplification and bisulfite sequencing was 73% (49/67).

Human Methylation 450k BeadChip (Illumina Inc.) was used for verification of the results in 10 nurse pairs of same age from the high- and low work stress environments. Fully methylated and unmethylated, bisulfite converted, DNAs (Epitech) and two duplicates were included for quality controls. DNA methylation data was then processed using GenomeStudio software (ver. 2011.0, Illumina Inc.). One sample, including its matched pair, was omitted from subsequent analyses due to incomplete bisulfite conversion.

### 5-HTTLPR Genotyping

*5-HTTLPR* genotype was assayed in two stages. Amplification of *5-HTTLPR* was carried out using PCR using the primers HTTLPR_F (gttgccgctctgaatgccag) and HTTLPR_R (ggataatgggggttgcaggg) that amplify either the long, L allele (280 bp product) or the short, S allele (236 bp product). The reaction was run in a total volume of 20 µl containing 60 ng of genomic DNA, 500 nM primers, 10 mM dNTP, 2.5 mM MgCl_2_ and 0.7U Dynazyme II Hotstart polymerase (Finnzymes). Thermocycling conditions were 94°C initial denaturing for 10 min followed by 35 cycles of the following: denaturing at 94°C for 15 s, annealing at 65°C for 20 s and extension at 72°C for 25 s. This was followed by a final extension at 72°C for 10 min. 5 µl of pcr product was then mixed with 8 units of *Msp* I restriction enzyme (Pharmacia Biotech) in One-Phor-All assay buffer. The reaction mixture was then incubated at 37°C for 16 hours followed by 20 min at 65°C. 5-HTTLPR genotypes were assessed on a 2% gel.

### Statistical Analysis

Differences in mean MBI-GS and BDI scores between high- and low work stress groups were tested by the Student’s t-test. Correlations between methylation levels in all five CpG residues were calculated using the Pearson’s Correlation. Structural equation measurement model was analyzed in order to test the hypothesis that these correlations could be explained by a single latent factor. Principal component factor analysis was used to obtain the single sum methylation value METsum. Statistical significance (2-tailed p<0.05) between methylation of each five CpG residue and work stress was assessed using the Student’s t-test. Associations between work stress, MBI-GS, METsum and *5-HTTLPR* were assessed by analysis of covariance (ANCOVA). In the initial multivariable model, we analyzed the main effects of 6 variables. Non-significant variables were then systematically eliminated using a stepwise backward elimination method until all significant variables were left in the final model (see Table S1).

Goodness of fit by chi-squared test was used for examining differences between direct sequencing and 450 k BeadChip methylation results in the age-matched pairs.

The tools used in all analysis were SPSS Statistics 18, PLINK (http://pngu.mgh.harvard.edu/purcell/plink/) [43] and SAS analytics.

## Supporting Information

Figure S1 **Flow chart of sample selection**. A large cohort of 5615 health care professionals was divided into lowest and highest quartile on workplace aggregated stress as per Karasek Model. 422 nurses were invited for further analysis. 99 nurses attended to laboratory examination and successful measurements were available for 95 individuals Laboratory measurements were available from 95 individuals. After exclusion criteria, the promoter region of *SLC6A4* of 49 individuals was successfully amplified and bisulfite sequenced. Success of sequencing was based on DNA yield after bisulfite conversion and PCR amplification.
(TIF)

Figure S2 **Distribution** **of methylation levels in the high and work stress environments.** Work stress is defined according to Karasek’s Model by the difference between averages of work demand and control.
(TIF)

Table S1 A. METsum in high and low work stress groups and work stress environment as a whole. The effect size can be calculated using Cohen’s d, defined as the difference between the two means of high and low work stress groups divided by the standard deviation for the complete data. B. Results of the initial main effects model using METsum as dependent variable.
(DOC)

## References

[1] Folkman S, Lazarus RS, Dunkel-Schetter C, DeLongis A, Gruen RJ (1986) Dynamics of a stressful encounter: cognitive appraisal, coping, and encounter outcomes. J Pers Soc Psy 50(5): 992–1003. 10.1037//0022-3514.50.5.992 3712234

[2] McEwen BS (1998) Protective and damaging effects of stress mediators. N Eng J Med 338: 171–9. 10.1056/NEJM199801153380307 9428819

[3] Maslach C, Schaufeli WB, Leiter MP (2001) Job burnout. Ann Rev Psy 52: 397–422. 10.1146/annurev.psych.52.1.397 11148311

[4] Uchida S, Hara K, Kobayashi A, Otsuki K, Yamagata H, et al (2011) Epigenetic status of Gdnf in the ventral striatum determines susceptibility and adaptation to daily stressful events. Neuron. 69(2): 259–72. 10.1016/j.neuron.2010.12.023 21262472

[5] McGowan PO, Sasaki A, D’Alessio AC, Dymov S, Labonté B, et al. 2009. Epigenetic regulation of the glucocorticoid receptor in human brain associates with childhood abuse. Nat Neurosci 12(3): 342–348. 19234457 10.1038/nn.2270 PMC2944040

[6] Bell JT, Pai AA, Pickrell JK, Gaffrey DJ, Pique-Regi R, et al (2011) DNA methylation patterns associate with genetic and gene expression variation in HapMap cell lines. Genome Biol 12: R10. 21251332 10.1186/gb-2011-12-1-r10 PMC3091299

[7] Murgatroyd C, Patchev AV, Wu Y, Micale V, Bockmühl Y, et al (2009) Dynamic DNA methylation programs persistent adverse effects of early-life stress. Nat Neurosci 12: 1559–1566. 19898468 10.1038/nn.2436

[8] Weaver ICG, Cervoni N, Champagne FA, D’Alessio AC, Sharma S, et al (2004) Epigenetic programming by maternal behavior. Nat Neurosci 7: 847–854. 15220929 10.1038/nn1276

[9] Devlin AM, Brain U, Austin J, Oberlander TF (2010) Prenatal exposure to maternal depressed mood and the MTHFR C677T variant affect SLC6A4 methylation in infants at birth. PLoS ONE 16: 5 (8).. 10.1371/journal.pone.0012201 PMC2922376 20808944

[10] Beach SR, Brody GH, Todorov AA, Gunter TD, Philibert RA (2010) Methylation at SLC6A4 is linked to family history of child abuse: an examination of the Iowa adoptee sample. Am J Med Genet B Neuropsyhiatr Genet 153B: 710–713. 10.1002/ajmg.b.31028 PMC2909112 19739105

[10] Beach SR, Brody GH, Todorov AA, Gunter TD, Philibert RA (2011) Methylation at 5HTT mediates the impact of child sex abuse on women’s antisocial behavior: an examination of the Iowa adoptee sample. Psychosom Med 73(1): 83–7. 20947778 10.1097/PSY.0b013e3181fdd074 PMC3016449

[12] Koenen KC, Uddin M, Chang SC, Aiello AE, Wildman DE, et al (2011) SLC6A4 methylation modifies the effect of the number of traumatic events on risk for posttraumatic stress disorder. Depress Anxiety 28(8): 639–647. 21608084 10.1002/da.20825 PMC3145829

[13] Philibert R, Madan A, Andersen A, Cadoret R, Packer H, et al (2007) Serotonin transporter mRNA levels are associated with the methylation of an upstream CpG island. Am J Med Genet B Neuropsychiatr Genet 144: 101–105. 10.1002/ajmg.b.30414 16958039

[14] Jirtle RL, Skinner MK (2007) Environmental epigenomics and disease susceptibility. Nat Rev Genet. 8(4): 253–62. 10.1038/nrg2045 PMC5940010 17363974

[15] Tsankova NM, Berton O, Renthal W, Kumar A, Neve RL, et al (2006) Sustained hippocampal chromatin regulation in a mouse model of depression and antidepressant action. Nat Neurosci 9(4): 519–25. 16501568 10.1038/nn1659

[16] Meyer-Lindenberg A (2009) Neural connectivity as an immediate phenotype: brain networks under genetic control. Hum Brain Mapp 30: 1938–1946. 19294651 10.1002/hbm.20639 PMC6871064

[17] Jans LA, Riedel WJ, Markus CR, Blokland A (2007) Serotonergic vulnerability and depression: assumptions, experimental evidence and implications. Mol Psychiatry 12(6): 522–43. 17160067 10.1038/sj.mp.4001920

[18] Conroy J, Meally E, Kearney G, Fitzgerald M, Gill M, et al (2004) Serotonin transporter gene and autism: a haplotype analysis in an Irish autistic population. Mol Psychiatry 9: 587–593. 14708029 10.1038/sj.mp.4001459

[19] Gorwood P (2004) Eating disorders, serotonin transporter polymorphisms and potential treatment response. Am J Pharm 4(1): 9–17. 10.2165/00129785-200404010-00002 14987118

[20] Lotrich FE, Pollock BG (2004) Meta-analysis of serotonin transporter polymorphisms and affective disorders. Psychiatr Genet 14(3): 121–129. 15318024 10.1097/00041444-200409000-00001

[21] Caspi A, Sugden K, Moffitt TE, Taylor A, Craig IW, et al (2003) Influence of Life Stress on Depression: Moderation in the 5-HTT Gene. Science 301: 386–389. 12869766 10.1126/science.1083968

[22] Chorbov VM, Lobos EA, Todorov AA, Heath AC, Botteron KN, et al (2007) Relationship of 5-HTTLPR genotypes and depression risk in the presence of trauma in a female twin sample. Am J Med Genet B 144: 830–833. 10.1002/ajmg.b.30534 17455215

[23] Lesch KP, Balling U, Gross J, Strauss K, Wolozin BL, et al (1994) Organisation of the human serotonin transporter gene. J Neural Trans. 95: 157–162. 10.1007/BF01276434 7865169

[24] Lesch KP, Bengel D, Heils A, Sabol SZ, Greenberg BD, et al (1996) Association of anxiety related traits with a polymorphism in the serotonin transporter gene regulatory region. Science 274: 1527–1531. 8929413 10.1126/science.274.5292.1527

[25] Hu X, Zhu G, Lipsky R, Goldman D (2004) HTTLPR allele expression is codominant, correlating with gene effects on fMRI and SPECT imaging intermediate phenotypes, and behavior. Biol Psychiatry 55: 191S.

[26] Kivimäki M, Lawlor DA, Smith GD, Kouvonen A, Virtanen M, et al (2007) Socioeconomic position, co-occurrence of behavior-related risk factors, and coronary heart disease: the Finnish Public Sector study. Am J Public Health 97: 874–9. 17395837 10.2105/AJPH.2005.078691 PMC1854863

[27] Karasek R (1998). Demand-Control Model: A social, emotional, and physiological approach to stress risk and active behaviour development. In J. M. Stellmann (Ed.), Encyclopaedia of Occupational Health and Safety, 4th Edn.

[28] van Ijzendoorn MH, Caspers K, Bakermans-Kranenburg J, Beach SRH, Philibert R (2010) Methylation matters: interaction between methylation density and serotonin transporter genotype predicts unresolved loss or trauma. Biol Psy 68: 405–407. 10.1016/j.biopsych.2010.05.008 PMC2921470 20591416

[29] Kinnally EL, Capitanio JP, Leibel R, Deng L, Leduc C, et al (2010) Epigenetic regulation of serotonin transporter expression and behavior in infant rhesus macaques. Genes Brain Behav 9: 575–582. 20398062 10.1111/j.1601-183X.2010.00588.x PMC2921011

[30] Shoemaker R, Deng J, Wang W, Zhang K (2010) Alelle-specific methylation is prevalent and is contributed by CpG-SNPs in the human genome. Genome Res 20(7): 883–889. 20418490 10.1101/gr.104695.109 PMC2892089

[31] Haenisch B, Bänisch H (2010) Depression and antidepressants: insights from knockout of dopamine, serotonin or noradrenaline re-uptake transporters. Pharmacol Ther. 129(3): 352–68. 10.1016/j.pharmthera.2010.12.002 21147164

[32] Berger M, Gray JA, Roth BL (2009) The Expanded Biology of Serotonin. Annu Rev Med 60: 355–66. 19630576 10.1146/annurev.med.60.042307.110802 PMC5864293

[33] Ruhe HG, Mason NS, Schene AH (2007) Mood is indirectly related to serotonin, norepinephrine and dopamine levels in humans: a metal-analysis of monoamine depletion studies. Mol Psychiatry 12: 331–59. 17389902 10.1038/sj.mp.4001949

[34] Han W, Cauchi S, Herman JG, Spivack SD (2006) DNA Methylation mapping by taq-modified bisulfite genomic sequencing. Analytical Biochem. 355: 50–61. 10.1016/j.ab.2006.05.010 16797472

[35] Shen L, Guo Y, Chen X, Ahmed S, Issa JP (2007) Optimizing annealing temperature overcomes bias in bisulfite PCR methylation analysis. Biotechniques 42: 48–58. 17269485 10.2144/000112312

[36] Breitling LP, Yang R, Korn B, Burwinkel B, Brenner H (2011) Tobacco-smoking-related differential DNA methylation: 27k discovery and replication. Am J Hum Genet 88: 450–457. 21457905 10.1016/j.ajhg.2011.03.003 PMC3071918

[37] Bönsch D, Lenz B, Reulbach U, Kornhuber J, Bleich S (2004) Homocysteine associated genomic DNA hypermethylation in patients with chronic alcoholism. J Neural Transm. 111: 1611–1616. 10.1007/s00702-004-0232-x 15565495

[38] Philibert RA, Sandhu H, Hollenbeck N, Gunter T, Adams W, et al (2008) The relationship of 5HTT (SLC6A4) methylation and genotype on mRNA expression and liability to major depression and alcohol dependence in subjects from the Iowa Adoption Studies. Am J Med Genet B Neuropsychiatr Genet 147B: 543–549. 17987668 10.1002/ajmg.b.30657 PMC3643119

[38] Schaufeli WB, Leiter MP, Maslach C, Jackson SE (1996). The MBI-General Survey. In C. Maslach, S.E. Jackson, & M.P Leiter (Eds.), Maslach Burnout Inventory. Manual (3rd ed., 19–26). Palo Alto, CA: Consulting Psychologists Press.

[39] Beck AT, Ward C, Mendelson M (1961) Beck Depression Inventory (BDI). Arch Gen Psychiatry 4: 561–571. 13688369 10.1001/archpsyc.1961.01710120031004

[40] Beck AT, Steer RA, Garbin MG (1988) Psychometric properties of the Beck depression inventory: twenty-five years of evaluation. Clin Psychol Rev 8: 77–100.

[41] Frommer M, McDonald LE, Millar DS, Collis CM, Watt F, et al (1992) A genomic sequencing protocol that yields a positive display of 5-methylcytosine residues in individual DNA strands. Proc Natl Acad Sci U S A 1 89(5): 1827–31. 10.1073/pnas.89.5.1827 PMC48546 1542678

[42] Lewin J, Schmitt AO, Adorjan P, Hildmann T, Pieperbrock C (2004) Quantitative DNA methylation analysis based on four-dye trace data from direct sequencing of PCR amplificates. Bioinformatics 20(17): 3005–12. 15247106 10.1093/bioinformatics/bth346

[43] Purcell S, Neale B, Todd-Brown K, Thomas L, Ferreira MAR, et al (2007) PLINK: a toolset for whole-genome association and population-based linkage analysis. American Journal of Human Genetics 81: 559–575. 17701901 10.1086/519795 PMC1950838

