# Supplementary figures and images for: Environmental Stress Affects DNA Methylation of a CpG Rich Promoter Region of Serotonin Transporter Gene in a Nurse Cohort
Source: PLoS One. 2012 Sep 28;7(9):e45813. doi: 10.1371/journal.pone.0045813 (PMC3461019; doi:10.1371/journal.pone.0045813)

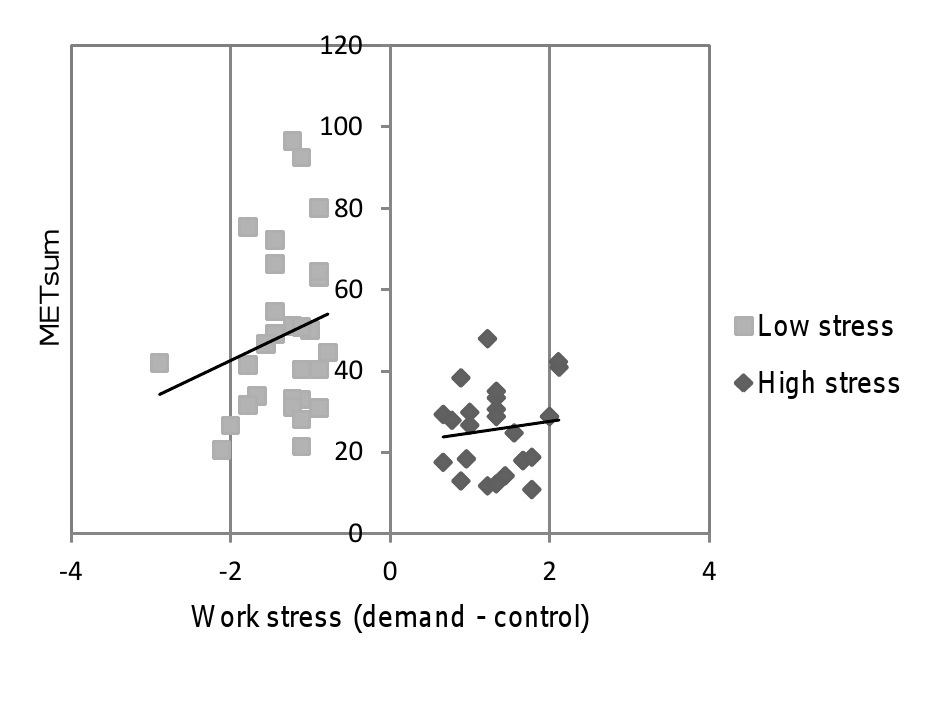

Supplement: Figure S2 — Distribution of methylation levels in the high and work stress environments. Work stress is defined according to Karasek’s Model by the difference between averages of work demand and control. (TIF) [file pone.0045813.s002.tif]
