# Supplementary material for: Environmental Stress Affects DNA Methylation of a CpG Rich Promoter Region of Serotonin Transporter Gene in a Nurse Cohort
Source: PLoS One. 2012 Sep 28;7(9):e45813. doi: 10.1371/journal.pone.0045813 (PMC3461019; doi:10.1371/journal.pone.0045813)
Supplement: Table S1 — A. METsum in high and low work stress groups and work stress environment as a whole. The effect size can be calculated using Cohen’s d, defined as the difference between the two means of high and low work stress groups divided by the standard deviation for the complete data. B. Results of the initial main effects model using METsum as dependent variable. (DOC) [file pone.0045813.s003.doc]

**Tables S1: A.** **METsum in high and low work stress groups and work stress environment as a whole.** The effect size can be calculated using Cohen’s d, defined as the difference between the two means of high and low work stress groups divided by the standard deviation for the complete data. **B.** **Results of the initial main effects model using METsum as dependent variable.**

**A.**

| **Work stress environment** | **Mean** | **SD** | **Lower 95% CL for Mean** | **Upper 95% CL for Mean** |
| --- | --- | --- | --- | --- |
| High work stress | 50.7 | 21.0 | 42.0 | 59.4 |
| Low work stress | 25.7 | 10.6 | 21.2 | 30.1 |
| Work stress environment | 38.4 | 20.8 | 32.5 | 44.4 |

**B.**

| **Variable** | **df** | **Type III SS** | **F** | **P** |
| --- | --- | --- | --- | --- |
| Work stress | 1 | 1480.196769 | 5.66 | 0.0221 |
| 5-HTTLPR | 2 | 685.987277 | 1.31 | 0.2806 |
| MBI-GS | 1 | 695.005206 | 2.66 | 0.1108 |
| Control | 1 | 222.192198 | 0.85 | 0.3621 |
| Demand | 1 | 0.01083 | 0 | 0.9949 |
| Age | 1 | 448.159783 | 1.71 | 0.1979 |
